# Clinical significance of fragmented QRS complexes or J waves in patients with idiopathic ventricular arrhythmias

**DOI:** 10.1371/journal.pone.0194363

**Published:** 2018-04-25

**Authors:** Choong Sil Seong, Hye Bin Gwag, Jin Kyung Hwang, Seung Jung Park, Kyoung-Min Park, June Soo Kim, Young Keun On

**Affiliations:** Division of Cardiology, Department of Internal Medicine, Heart Vascular and Stroke Institute, Samsung Medical Center, Sungkyunkwan University School of Medicine, Seoul, Republic of Korea; University of Tampere, FINLAND

## Abstract

**Background:**

Idiopathic ventricular fibrillation (IVF) can cause sudden cardiac death. Previous studies have reported that J waves and fragmented QRS complexes (f-QRS) are arrhythmogenic markers and predictors of cardiac events. We evaluated the prevalence and clinical significance of J waves and f-QRS in patients with IVF.

**Methods:**

We studied 81 patients who received an implantable cardioverter defibrillator (ICD) due to IVF between October 1999 and June 2015. We assessed the prevalence of J waves and f-QRS using electrocardiograms (ECGs). Patients were classified into three groups: J wave group (n = 35), f-QRS group (n = 20), or normal ECG group (n = 26). The control group included 81 subjects without heart disease who were matched for age, sex, and race. We compared syncope, sudden cardiac arrest, and appropriate ICD shock between the three groups.

**Results:**

The follow-up duration was 4.1 years. J waves and f-QRS were more frequent in patients with IVF than in control subjects (43.2%, 21% vs. 24.7%, 19.7%, *P* < 0.001). Out of the three groups, clinical cardiac events were most frequent in the f-QRS group (50% vs. 45.7% vs. 11.5%, *P* = 0.028). A comparison of the combined group of J wave and f-QRS versus the normal ECG group revealed that the combined group had a higher frequency of clinical cardiac events than the normal ECG group (47.3% vs. 11.5%, respectively, *P* = 0.009).

**Conclusions:**

Patients with IVF had higher prevalence of f-QRS or J waves. And patients with f-QRS or J waves were at higher risk of recurrent ventricular fibrillation.

## Introduction

Sudden cardiac death (SCD) is a major public health problem and accounts for 300,000 to 400,000 deaths annually in the United States.[1] Most cases of sudden cardiac death are caused by ventricular tachyarrhythmia such as ventricular tachycardia (VT) and ventricular fibrillation (VF).[2] Idiopathic ventricular fibrillation (IVF) without structural heart disease accounts for less than 5% of all cases of SCD.[3] Previous studies have suggested that J waves and fragmented QRS complexes (f-QRS) play critical roles in the pathogenesis of IVF.[4–8]

Early repolarization is a common electrocardiographic finding. The J point is the point at which the QRS complex joins the ST-segment. The J wave (J-point elevation) is also known as the camel hump sign, the late delta wave, the J point wave, the hat-hook junction, the K wave, the H wave, and the Osborn wave including early repolarization.[9] Although early repolarization is usually considered benign, some evidence suggests that it can potentially lead to arrhythmogenicity.[10] Previous clinical studies have demonstrated that patients with a history of IVF have increased rates of early repolarization.[4–6]

The f-QRS on 12-lead ECG have been suggested as a marker of myocardial scarring associated with arrhythmic events in coronary artery disease and non-ischemic cardiomyopathy.[11, 12] Previous studies have also reported that f-QRS is a predictor of cardiac events and mortality in patients with structural heart disease.[13–18]

The f-QRS complexes and J waves were identified variously from general population to patients with structural heart disease. In one study, the prevalence of J waves in 1817 healthy subjects was 7.26%.[19] In another general population study, the prevalence of J waves were 5.8%[20] and 13.1%[21]. In 152 patients with an ST-elevation myocardial infarction (MI) after percutaneous coronary intervention (PCI), the prevalence of J waves was 60.5%.[22] The f-QRS complexes were identified in 5.1% of 1500 consecutive healthy adults.[23] The f-QRS complexes were identified in 35% of 479 patients with CAD, in 51% of 105 patients with non-ischemic cardiomyopathy, in 85% of 360 patients with ARVD, and in 43% of 115 patients with Brugada syndrome.[7]

The 12-lead ECG is an easily obtainable graph for observing J waves and f-QRS, both of which are potentially useful predictors of ventricular tachyarrhythmia. The J wave has been shown to be associated with repolarization abnormalities, whereas f-QRS has been associated with depolarization abnormalities. However, few studies have focused on the presentation of J waves and f-QRS in patients with IVF.[24] The aim of this study was to evaluate the prevalence and clinical significance of J waves and f-QRS in patients with IVF but without structural heart disease.

## Methods

### Study population

**Fig 1:**
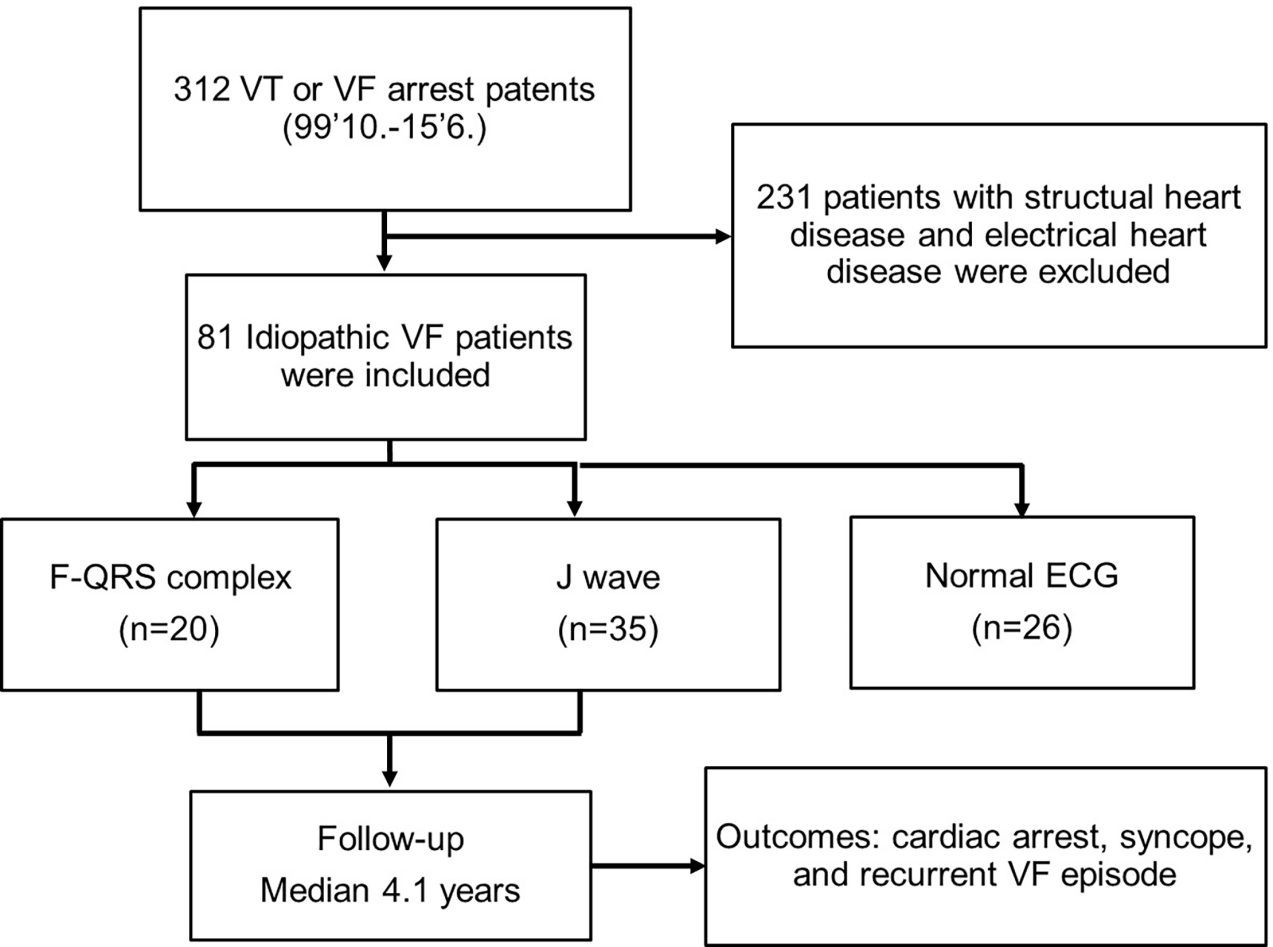
(A) Flow chart of study subjects.

We included 312 patients who had received an implantable cardioverter defibrillator (ICD) due to ventricular tachyarrhythmia between October 1999 and June 2015 at the Samsung Medical Center. We enrolled 81 patients with IVF and excluded patients with structural heart disease or electrical heart disease (Fig 1).

### Data collection

We assessed the prevalence of J wave and f-QRS on resting 12-lead electrocardiograms (ECG). Patients were classified into three different groups based on their electrocardiographic morphology: J wave group (group I), f-QRS group (group II), and normal ECG group (group III). ECGs from patients in group III did not contain J waves or f-QRS. We compared patient baseline characteristics, history of syncope, sudden cardiac arrest, and appropriate ICD shock between the three groups. We also compared the prevalence of J waves and f-QRS to those of the control group. We selected control group without heart disease who were matched for age, sex, and race as random. The control group was those who visited the department of cardiology and had no abnormality on the cardiac examination and tests.

Patients were classified as having IVF if they had no detectable structural heart disease by echocardiography, coronary angiography (CAG). But we performed magnetic resonance imaging (MRI) in limited patients, only 34 subjects. We excluded patients with electrical heart diseases, such as Brugada syndrome, catecholamine-induced polymorphic ventricular tachycardia, or congenital long- and short-QT syndromes. We performed genetic testing for patients with suspected of Brugada syndrome or Long QT syndrome. Patients who were strongly suspected of electrocardiogram or who were diagnosed with genetic testing were excluded.

### ECG analysis for J waves and f-QRS

The 12-lead ECGs were evaluated for the presence of J waves and f-QRS. Two cardiologists independently reviewed each ECG. Standardized ECG filter is used for all tests. J waves were defined as QRS slurring or notching associated with an elevation of the QRS-ST junction (J point) elevation in at least two contiguous leads on the resting 12-lead ECG. The amplitude of J-point elevation had to be at least 1 mm (0.1mV) above the baseline level, either as QRS slurring or notching in the inferior leads (II, III, and aVF), lateral leads (I, aVL, and V4 to V6), or both.[5] The anterior precordial leads (V1 to V3) were not analyzed in order to exclude patients with arrhythmic right ventricular dysplasia (ARVD) or Brugada syndrome. We also classified early repolarization in the J-wave group into 4 types: (1) QRS slurring without ST elevation, (2) QRS notching without ST elevation, (3) QRS slurring with ST elevation, and (4) QRS notching with ST elevation.[25]

f-QRS was defined as the presence of an additional R wave (R’), a notching in the nadir of the R wave or the nadir of the S wave, or the presence of more than one R prime (fragmentation) in two contiguous leads, corresponding to a major coronary artery territory. Typical bundle branch block (BBB) pattern (QRS ≥ 120 ms) and incomplete right BBB (100 < QRS < 120 ms) in V1 or V2 were excluded from analysis.[11] We determined the presence of f-QRS in ≥ 2 contiguous precordial leads (V1 to V5), in ≥ 2 contiguous lateral leads (I, aVL, and V6), and in ≥ 2 contiguous inferior leads (II, III, and aVF). The f-QRS included various RSR’ patterns (RSR’, rSr’, rSR’, notched S, notched R, and fragmented QRS).

### Follow-up and data collection

All patients received an implantable defibrillator and were followed-up in the outpatient clinic every 3 to 6 months. The following clinical data were collected: history of unexplained syncope, circumstances of sudden cardiac arrest, familial history of SCD, and rate of recurrent VF or syncope. Recurrence was defined as documented episodes of VF or VT, followed by appropriate ICD shocks including syncope, cardiac arrest, or SCD. Follow-up cardiac events were collected based on appropriate shocks recorded by the ICD and patient symptoms. Twelve-lead ECG, chest X-ray, and echocardiography was repeated.

### Statistical analysis

Continuous variables were reported as the mean ± standard deviation (SD), whereas categorical variables were reported as the percentage in each group. Continuous variables were compared with Student’s *t*-test. Categorical variables were compared with the chi-square test or Fisher’s exact test. Kaplan-Meier curves stratified by the three groups were generated to analyze the outcomes of recurrent clinical cardiac events using the log-rank test. *P* values < 0.05 were considered significant.

### Ethics statement

This study protocol was reviewed and approved by the institutional review board of the Samsung Medical Center (IRB No. SMC 2017-09-091). The patient records used in our retrospective study. We had all data fully anonymized and the IRB committee waived the requirement for informed consent.

## Results

### Baseline characteristics

The patient baseline clinical characteristics are shown in Table 1. Group I (f-QRS group) consisted of 20 patients (mean age 42.2 ± 18.3 years, 80% males), Group II (J wave group) consisted of 35 patients (mean age 37.5 ± 16.4 years, 91.5% males), and group III (normal ECG group, no J waves or f-QRS) consisted of 26 patients (mean age 30.4 ± 15.6 years, 57.7% males). Significant differences in sex were observed between the three groups; males comprised the majorities in groups I and II (80.0% and 91.5%, respectively). Patients in the normal ECG group were younger than those in the other groups. There were no significant differences in any of the other baseline characteristics analyzed.

**Table 1:** Patient baseline characteristics and clinical cardiac events.

	f-QRS (n = 20)	J wave (n = 35)	Normal ECG (n = 26)	*P* value
Age, years	42 ± 18	36 ± 16	30 ± 16	0.597
Male, n (%)	16 (80.0%)	32 (91.5%)	15 (57.7%)	0.007
Body mass index, kg/m^2^	22 ± 3	22 ± 3	23 ± 4	0.607
Diabetes mellitus	3 (15%)	2 (6%)	3 (12%)	0.509
Hypertension	4 (20%)	4 (11%)	5 (19%)	0.612
Chronic kidney disease	0 (0%)	2 (6%)	0 (0%)	0.260
Current smoker	6 (30%)	6 (17%)	8 (31%)	0.245
Family History of SCD	1 (5%)	5 (14.3%)	1 (3.8%)	0.751
History of CVA	0 (0%)	1 (2.9%)	0 (0%)	0.514
Recurrent VF episode	10 (50%)	16 (45.7%)	3 (11.5%)	0.028
Recurrent VF episode (J wave group combined with f-QRS group)	26 (47.3%)		3 (11.5%)	0.009
Cardiac death	0	0	0	

The values are presented as mean ± SD or number of patients (%).

f-QRS, fragmented QRS complexes; SCD, sudden cardiac death; CVA, cerebrovascular accident; VF, ventricular fibrillation; ICD, Implantable cardioverter defibrillator

Normal ECG, neither f-QRS nor J wave

Clinical cardiac events = cardiac arrest, syncope, and recurrent VF episode recorded in ICD

### Prevalence of f-QRS and J waves

The prevalence of f-QRS and J waves on each contiguous lead compared to those of the matched-control group are shown in Table 2. The prevalence of f-QRS in any lead among patients with IVF was 20 out of 81 patients (24.7%). The prevalence of f-QRS in the inferior leads, lateral leads, and anterior leads were 60%, 20%, and 20%, respectively (Table 2). The prevalence of f-QRS in the control group was 16 out of 81 patients (19.7%). f-QRS presented more frequently in patients with idiopathic VF than in the matched-control group (24.7% vs. 19.7%, respectively).

**Table 2:** Incidences of f-QRS and J waves in patients with IVF and matched -control subjects.

	Patients with IVF			Matched–control subjects		
	f-QRS *	J wave †	*P* value	f-QRS	J wave	*P* value
Any leads	20 (24.7%)	35 (43.2%)	< 0.001	16 (19.7%)	17 (21.0%)	< 0.001
Inferior leads	12 (60%)	25 (71.4%)		10 (62.5%)	12 (70.6%)	
Lateral leads	4 (20%)	8 (22.9%)		0 (0%)	3 (17.6%)	
Anterior leads	4 (20%)	-		6 (37.5%)	-	
Both Inferior and lateral leads	-	2(5.7%)		-	2 (11.8%)	

The values are presented as number of patients (%).

f-QRS, fragmented QRS complexes; IVF, idiopathic ventricular fibrillation

* f-QRS was identified in inferior leads (II, III, and aVF), lateral leads (I, aVL, and V6), and anterior leads (V1-5)

† J-wave was identified in inferior leads (II, III, and aVF), lateral leads (I,aVL, and V4 to V6), or both

J waves in any leads were identified in 35 of the 81 patients with IVF (43.2%). The prevalence of J waves in the inferior leads, lateral leads, and both leads were 71.4%, 22.9%, and 5.7%, respectively. In the matched-control group, J waves were identified in 17 of the 81 patients (21.0%). J waves presented more frequently in patients with idiopathic VF than in the matched-control group (43.2% vs. 21.0%). We identified the 4 types of early repolarization in the J wave group: (1) QRS slurring without ST elevation (9 patients), (2) QRS notching without ST elevation (13 patients), (3) QRS slurring with ST elevation (2 patients), and (4) QRS notching with ST elevation (11 patients).

Our data indicated that f-QRS and J waves were both more frequent in patients with IVF than in control patients. Moreover, both f-QRS and J waves presented most commonly in contiguous inferior leads (60% and 71.4%, respectively).

**Fig 2:**
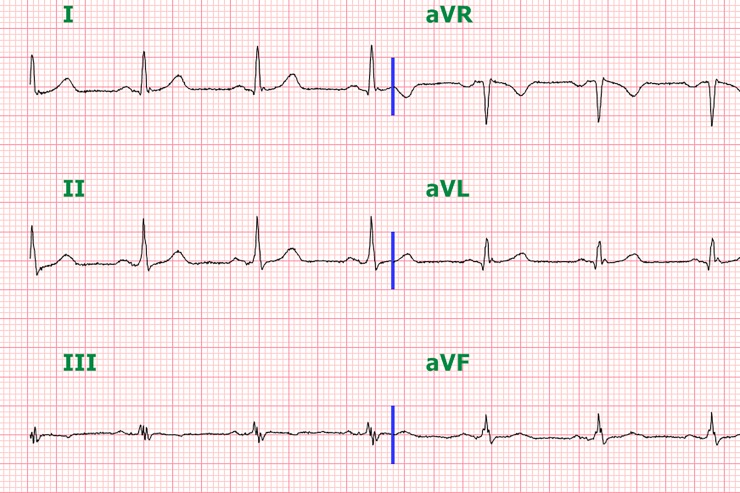
ECG of patient with both f-QRS (III, aVF leads) and J wave (I, aVL leads).

Three patients had both f-QRS and J waves concomitantly. They were 71-, 46-, and 56-year-old males. Both f-QRS and J waves were seen in the inferior leads in two patients. In one patient, the f-QRS was seen in the inferior leads and the J waves were in lateral leads (Fig 2). We could not identify specific baseline characteristics and correlation with MACEs due to the small sample size.

### Therapy and follow-up

All patients with IVF received an implantable cardioverter defibrillator (ICD). Patients took oral antiarrhythmic agents during the follow-up period, including amiodarone, flecainide, propafenone, beta-blockers (BB), and calcium channel blocker (CCB). Many patients didn’t take medications and the patients who had received medication mostly took beta-blockers: 20 patients in the f-QRS group [no medications (7), BB (5), CCB (2), amiodarone (1), CCB + BB (2), amiodarone + BB (2), and flecanide + BB (1)], 35 patients in the J wave group [no medications (15), BB (13), CCB (2), amiodarone (2), CCB + BB (1), propafenone (1), and propafenone + BB (1)], and 26 patients in the normal ECG group [no medications (16), BB (9), and amiodarone (1)].

**Fig 3:**
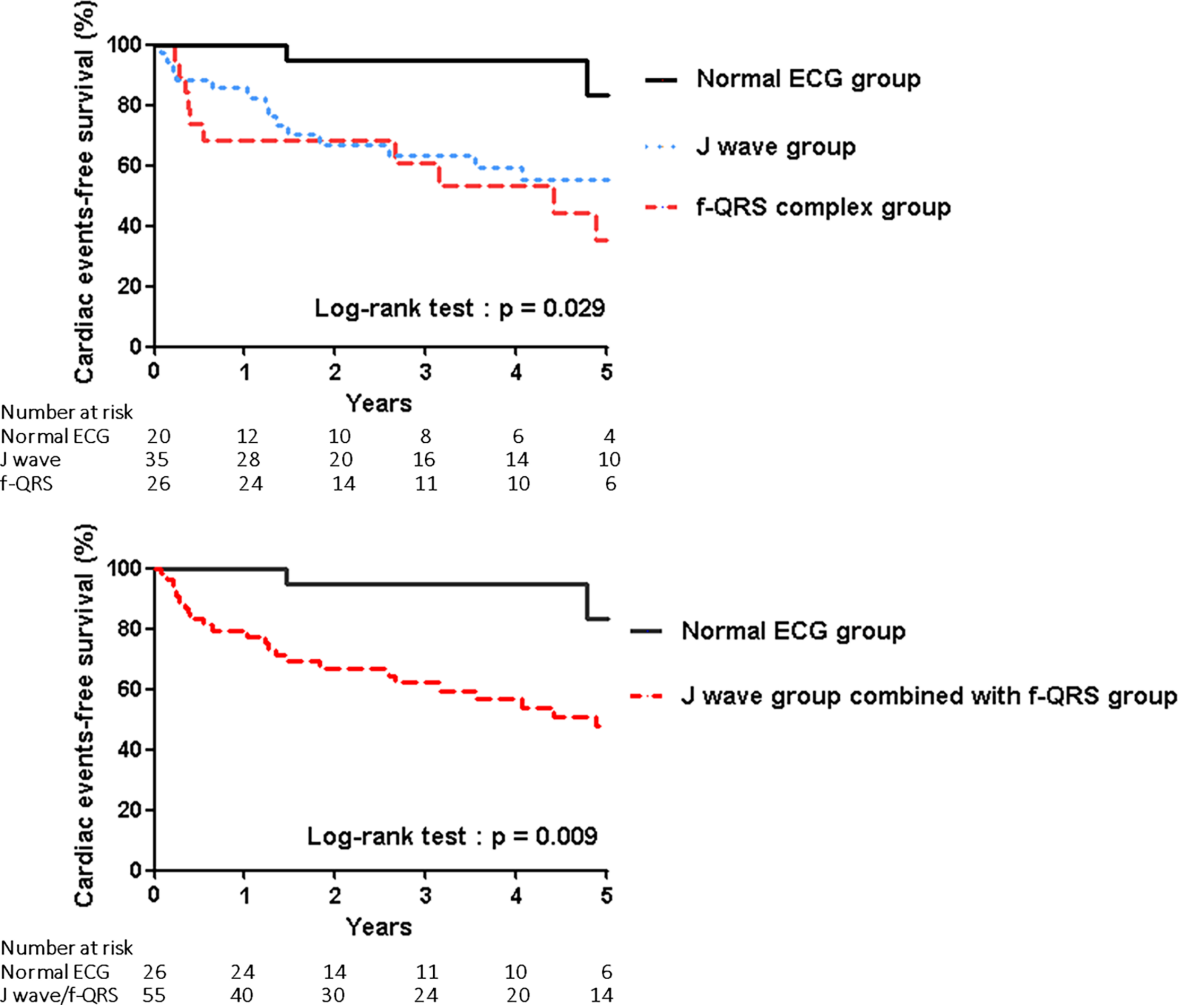
(A) Kaplan-Meier curves of clinical cardiac events in the three groups. (B) Kaplan-Meier curves of clinical cardiac events in the J wave group combined with f-QRS group versus the normal ECG group.

We successfully obtained information on VF recurrence. The median follow-up duration was 4.1 years. Clinical cardiac events (cardiac death, syncope, and VF episodes recorded by the ICD) were more frequent in the f-QRS and J waves group than in the normal ECG group (50% vs 45.7 vs 11.5%, respectively, *P* = 0.028) (Table 1). Moreover, the prevalence of clinical cardiac events in the J wave group combined with the f-QRS group versus those in the normal ECG group were significantly different (47.3% vs. 11.5%, *P* = 0.009). Kaplan-Meier curves of clinical cardiac events in the three groups and in the J wave group combined with the f-QRS group versus the normal ECG group are shown in Fig 3. The majority of clinical cardiac events were recurrence of VF episodes recorded by the ICD. No cardiac deaths occurred in any of the groups.

## Discussion

Sudden cardiac arrest has been commonly associated with ventricular tachyarrhythmia. Malignant ventricular tachyarrhythmia such as VT and VF are known to occur in individuals with structural heart disease (e.g., coronary disease, cardiomyopathy, or heart failure) or electrical heart disease (e.g., long/short QT syndrome or Brugada syndrome). SCD has also been shown to occur in patients with IVF without structural heart disease or electrical heart disease. Arrhythmogenicity of f-QRS and J waves has been reported in patients with IVF. Here, we studied both f-QRS and J waves in patients with IVF. This study presented new information on the prevalence, clinical characteristics, and prognosis of patients with IVF and f-QRS or J waves. f-QRS and J waves were more commonly identified in patients with IVF than in control patients. Moreover, patients with f-QRS or J waves suffered more recurrent VF episodes compared to patients without f-QRS or J waves. This study indicates that patients with IVF have an increased prevalence of f-QRS or J wave, and patients with f-QRS or J waves have higher risk of ventricular tachyarrhythmia.

Previous studies have suggested that f-QRS represents a marker of depolarization abnormality, i.e. conduction delay caused by the myocardial scar, as shown in f-QRS in body surface ECG.[13] A recent meta-analysis from twelve studies of f-QRS concluded that f-QRS is associated with all-cause mortality and the occurrence of SCD in both coronary artery disease and non-ischemic cardiomyopathy.[8] f-QRS has also been reported as a diagnostic marker in arrhythmogenic right ventricular dysplasia (ARVD)[26] and a prognostic indicator in patients with Brugada syndrome.[27] Although several studies have reported relationships between f-QRS and structural heart diseases, few studies have focused on patients diagnosed with IVF. Wang *et al*.[24] had described two hypotheses to explain the presence of f-QRS in patients with IVF: (1) sporadic scars in the ventricular myocardium that cannot be detected by echocardiography or magnetic resonance imaging and (2) a special physiological phenomenon, functional modulation of conduction caused by autonomic nerve activity, aging, temperature, or heart rate, based on previous studies about Brugada syndrome. Our data showed that patients with IVF had higher rate of f-QRS than control patients, even though they do not have structural heart disease. Moreover, f-QRS was particularly prevalent in the inferior leads as compared to the anterior and lateral leads. Patients with IVF and f-QRS had the highest rates of recurrent VF as compared to patients without f-QRS (groups II and III). In patients with IVF, f-QRS may be a suitable marker of SCD risk, as f-QRS is present in patients with structural heart disease.

Although J-point and ST-segment elevation in the right precordial leads (as is observed in Brugada syndrome) is considered to be a marker of arrhythmic risk in patients without structural heart disease, J-point and ST segment elevation in the lateral leads or inferior leads is considered to be a benign finding in young, healthy individuals. However, recent studies have reported that J waves (J-point elevation) are found more frequently and are considered to be a predictor of recurrent VF in patients with IVF.[4–6] J waves presented more commonly in the inferior leads and that patients with IVF were more likely to be male. We found that patients with IVF had a higher prevalence of J waves as compared to control patients and that J waves were more frequent in the inferior leads than in the other leads. These results were similar to those in a previous study. We also found that patients with J waves had a higher risk of recurrent VF than patients without J waves, which was based on VF episodes recorded by the ICD.

f-QRS represents a depolarization abnormality, while J waves represent a repolarization abnormality. We found that the risk of recurrent VF was higher in the f-QRS group than in the J wave group, but this difference was not significant. Both the f-QRS and J wave groups had higher percentages of males (80%, 91.5%); furthermore, both f-QRS and J waves were present at higher rates in the inferior leads (60% and 71.4%, respectively). All patients with IVF underwent ICD implantation at our hospital. Since we monitored appropriate shocks as recorded by the ICDs, we were able to evaluate the exact recurrence of VF. No cardiac deaths occurred in this study. Although patients with IVF had no structural heart disease, our data indicated that special attention should be paid to patients with IVF and f-QRS or J waves, especially younger males with f-QRS or J waves presenting in the inferior leads.

This study did have some limitations. First, this study was a retrospective observational study at a single medical center. Study for f-QRS and J waves is rare in Korea, especially for patients with IVF, and a multi-center prospective study is needed. We suggest prospective registry study enrolling subjects with ECG abnormalities of f-QRS and J wave. We would like to collect the data for ICD implantation criteria according to ECG abnormalities for primary prevention. Second, only a small number of patients with IVF and their matched-controls were included. Because we focused only on patients that received ICD implantation and we monitored appropriate shock as recorded by the ICD, the number of enrolled patients with IVF was further limited. The prevalence of f-QRS a J wave in our control group was higher than other study population. We think that the number of subjects selected is not enough to reflect the general population. Third, we could not evaluate cardiac MRIs for all the study subjects due to the retrospective nature of the study. Cardiac MRIs for only 34 patients were evaluated. We could not evaluate association between f-QRS and myocardial scar or fibrosis properly. Furthermore, three patients exhibited J waves and f-QRS simultaneously. We think that those who had both f-QRS and J wave are at higher risk. But it was difficult to detect correlation with MACEs due to small number. Further studies are necessary in order to determine the significance of simultaneous J waves and f-QRS in patients with IVF.

## Conclusion

Twelve-lead ECGs are easily available and relatively inexpensive to obtain. Thus, these tools have the power to benefit treatment and prognosis by detecting f-QRS and J waves in patients with IVF. We found that patients with IVF have increased prevalence of f-QRS and J waves and IVF patients with f-QRS or J waves have a greater risk of recurrent VF.

## Supporting information

S1 File Raw data of patients with IVF. (XLSX)

## Abbreviations

IVF: Idiopathic ventricular fibrillation
VT: Ventricular tachycardia
VF: Ventricular fibrillation
f-QRS: Fragmented QRS complexes
ICD: Implantable cardioverter defibrillator
ECG: Electrocardiograms
SCD: Sudden cardiac death
CAG: Coronary angiography
MRI: Magnetic resonance imaging
MACE: Major adverse cardiac event
